# Changes in viable bacterial counts and physicochemical parameters of water used during live transportation of Pangasius catfish (*Pangasianodon hypophthalmus*) in Bangladesh

**DOI:** 10.5455/javar.2022.i570

**Published:** 2022-01-16

**Authors:** A. N. M. Rezvi Kaysar Bhuiyan, Md. Mubarack Hossain, Md. Naim Uddin, Md. Anwar Hossain, Md. Ismail Hossain, Md. Nurul Haider

**Affiliations:** 1 Department of Fisheries Technology, Bangladesh Agricultural University, Mymensingh 2202, Bangladesh.; 2 Biswas Agro-Fisheries and Hatchery Ltd., Mahbub Group of Industries, Trishal, Mymensingh, Bangladesh.

**Keywords:** Total bacterial count, live transportation, Pangasius catfish, physicochemical parameters, live fish, welfare

## Abstract

**Objective:**

The study was undertaken to assess the changes in viable bacterial counts and physicochemical parameters of water used during the live transportation of Pangasius catfish (*Pangasianodon hypophthalmus*). The correlations between the changing patterns of these parameters were also established.

**Materials and Methods:**

Water samples were collected every 2 h interval, plated onto agar plates for assesing viable bacterial counts. Physicochemical parameters, namely, water temperature, dissolved oxygen (DO), pH, and ammonia (NH_3_) concentration in the water were measured with a glass thermometer, DO test kit, pH test kit, and total NH_3_ measurement kit, respectively.

**Results:**

The viable bacterial counts increased significantly from 0 to 2 h in all the studied channels and remained almost similar up to the end of the supply channels. The water temperature was almost stable regardless of the supply channels and transportation period at around 30°C. The DO concentration and pH level decreased, and NH_3_ concentrations increased gradually in all the supply channels. The viable bacterial counts were inversely correlated to the DO levels and directly associated with the NH_3_ concentrations of the water used during the live transportation of Pangasius catfish.

**Conclusions:**

Gradual increase in viable bacterial counts and fluctuation in some vital physicochemical parameters with the duration of transportation indicated an unfavorable environment for the survival of Pangasius catfish.

## Introduction

Pangasius catfish, *Pangasianodon hypophthalmus*, is very popular with the fish farmers of Bangladesh due to its fast growth, ease of culture technique, high disease resistance, and ability to withstand a wide range of environmental conditions [1]. Consumers also prefer the fish due to its low market price, which has become one of the most important cultured species in Bangladesh [2]. Pangasius catfish aquaculture provides about 18.37% of total aquaculture production in Bangladesh [3]. As consumers prefer to buy live fish, the Pangasius catfish are commonly transported in live condition from the culture areas to the retail markets. In Bangladesh, fish in the live state are usually transported either by “open truck system” or “tank system,” sometimes with oxygenation [4]. In the case of the “open truck system,” a plastic or tarpaulin is typically placed on the truck bed to contain the water. The volume of water and the amount of fish carried depend on the truck’s size and capacity. In the case of “tank systems,” cheap plastic tanks having a capacity of around 1 m^3^ are usually used. The tanks are sometimes reinforced by an iron frame [4]. However, live Pangasius catfish are also transported from the farm to the markets in a van, rickshaw, truck, passenger bus, pickup, etc., by keeping them in plastic tanks. In general, Pangasius catfish are harvested in the afternoon, transported the following night, and reach their final retail destination in the morning. Deep tube-well water is used with or without the water exchange during transportation, depending on the distance, and duration of the final destination.

Several processes may occur during the live transportation of fish. First of all, carbon dioxide (CO2) is generated and accumulated in the transport-water, which may become life-threatening to the fish, as high concentrations of CO2 limit the oxygen supply system of fish [5]. CO2 gas is readily dissolved in water and becomes a weak acid. The nontoxic bicarbonate ions from this acid invade the blood and make the blood plasma more acidic, which has harmful consequences for the fish. The severity of these limitations is affected by water temperature, water pH, the loading density of fish, and time in transport. Toxic accumulations of ammonia (NH_3_) and CO2 can kill fish, thus limiting the loading density [5]. All these stress-related factors (so-called stressors) induce conditions in which fish’s ability to survive during live transportation is impaired; they cannot maintain a normal physiological state as the stressors adversely affect their welfare [6]. The degree of interruption in the physiological condition of fish or interruption in fish welfare is thus directly or indirectly related to different acute stressors (such as rough treatment within captivity, fluctuations in the vital water quality parameters, and limited conditioning) and/or chronic stressors (such as poor water quality for an extended period, improper stocking densities, improper diets, etc.) [7]. Bacteria in water can even make the water quality worse for fish during live transportation. They consume part of it, compete with fish for dissolved oxygen (DO), and release toxic metabolites [8,9]. Bacteria increase the (NH_3_) concentration in the carrying water and compete with fish for DO [8]. Initial sources of bacteria in the live fish transporting water are the fish itself (fish gills, skin, and intestine contain bacteria), transporting water, and sediments from the cultured ponds [10]. The quantitative and qualitative presence of microorganisms in fish bodies is directly related to the microbial composition of the aquatic environment from where they are harvested [11]. Thus, bacterial concentrations increase in different organs of fish with an increased bacterial concentration in the surrounding water in which they live [12].

During live transportation of Pangasius catfish, changes in skin color, speedy respiration, and behavioral changes like nervousness, etc., are seen in the fish. Mechanical abrasion is also a common consequence of handling and transportation [13]. All these abnormal external appearances of Pangasius catfish hamper its preference by consumers and lead to comparatively fewer demanded products of Pangasius catfish. It is generally believed that higher bacterial growth, higher NH_3_ concentration, and lower DO levels negatively influence all of these unacceptable features in Pangasius during live transportation. But, we do not know how all these biological and physicochemical parameters change with time during live transportation. Many questions remain: when does the DO level become critical to the fish’s survival? How do the concentrations of NH_3_ vary according to the transportation period? Does the accumulation of metabolic wastes enhance the bacterial regrowth or limit the DO level of the water? To answer these questions, it is essential to evaluate the changes in the viable counts of bacteria and the physicochemical properties of water used during the live transportation of Pangasius catfish through different supply channels. We assumed that bacterial counts would gradually increase with the transport time because of their regrowth in the water. We also hypothesized that accumulation and decomposition of the metabolic wastes would reduce the pH and DO levels and enhance the concentration of NH_3_ in the transport water used to transport the live Pangasius catfish. All these consequences will show a positive correlation between the bacterial count and NH_3_ concentration and a negative correlation between viable counts and the oxygen concentration of the water. So the present study was undertaken to verify the assumptions mentioned above. 

## Materials and Methods

### Ethical statement

Live Pangasius catfish (P. hypophthalmus) transporting water was used in this research, and therefore, no ethical approval is required. Water samples were collected from the plastic barrels containing live Pangas fish during transportation without disturbing or harming them. However, prior permission was taken from the owners’ explain the aims of this research work, and sub-samples were collected upon their authorization.

### Descriptions of the studied supply channels of the Pangasius catfish

**Figure 1.**
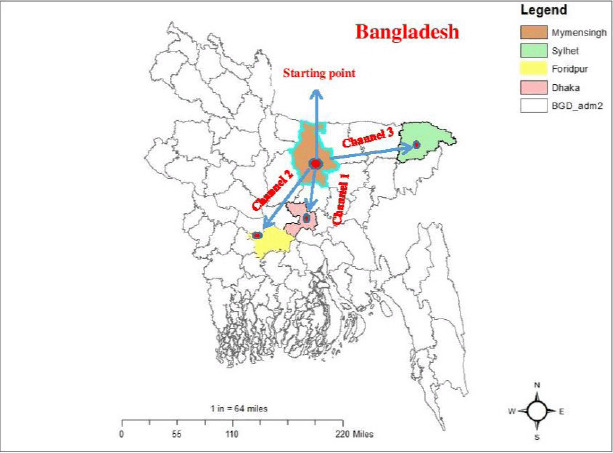
Map shows the location of sampling supply channels of live Pangasius catfish (P. hypophthalmus) transportation in Bangladesh. (Channel-1: Trishal, Mymensingh to Kawran Bazar, Dhaka; Channel-2: Trishal, Mymensingh to Faridpur; and Channel-3: Trishal, Mymensingh to Poschim Kazir Bazar, Sylhet). The map is extracted from DIVA-Geographical Information System (GIS) using GIS and visualized by ArcMap version 10.7.

From July to December 2019, samples were collected from three different Pangasius catfish supply channels in Bangladesh. All of the channels began in Trishal Upazila of Mymensingh district because it is one of Bangladesh’s major Pangasius catfish-producing areas. The supply channels were designated as “Channel 1” from Mymensingh to Dhaka, “Channel 2” from Mymensingh to Faridpur, and “Channel 3” from Mymensingh to Sylhet (Fig. 1).

### Harvesting and preparation before live transportation

The Pangasius catfish were harvested with the surrounding net and prepared for transportation at dusk. A very brief conditioning period was allowed before the transportation. In most cases, Pangasius catfish’s live transportation started at night and reached the final destination/unloading points (retail markets) at dawn. Around 40 kg of fish (20–22 fish) were loaded into a plastic-made transportation tank with 1,000 l. But the tanks were filled half with 500 l of deep tube-well water. Approximately 40–42 transportation tanks were incorporated into a commercial vehicle (truck). The exchange of water from the transportation tank with the deep tube-well water once every 2–3 h during the live transportation of Pangasius catfish was commonly done in all the supply channels during transportation. Pangasius catfish were collected from the transportation tank at the retail market, and live fish were separated from the dead ones.

### Collection of sub-samples for laboratory analyses

To collect the samples during transportation, necessary materials were carried and transported by the live Pangasius catfish transportation vehicle (truck). Culture experiments and assessment of physicochemical parameters were conducted onboard the vehicles; thus, prepared agar plates and test kits were also carried. Subsamples of water were collected from 0 h (at the beginning of transportation just after loading) until the end (reaching the retail markets/points before unloading) at every 2 h interval. Different transportation tanks were selected randomly, and water samples were collected in a sterilized plastic bottle (250 ml). After collecting water samples, subsequent analyses and cultures of bacteria for viable count were carried out immediately.

### Assessment of the viable bacterial count

The sample preparation and culture of bacteria for viable counts during live fish transportation was conducted according to Hossain et al. [8]. The collected water samples were diluted using sterilized physiological saline (0.85% W/V NaCl) to obtain the original sample’s 10^−1^, 10^−2^, 10^−3^, etc. From each of the above dilutions, 0.1 ml was taken aseptically to spread onto the previously prepared plate count agar plates (HIMEDIA, India). A replicate was maintained for each of the dilutions. The plates were then wrapped with aluminum foil and kept in zipper bags until they were finally brought to the Fisheries Microbiology Laboratory, Department of Fisheries Technology, Bangladesh Agricultural University, and incubated at 30°C for 48 h. After incubation, plates that exhibited 30–300 colonies were counted by a colony counter (Stuart Scientific Colony Counter, SC6PLUS-UK). The result of the viable bacterial count was expressed as the number of colony-forming units (CFU) per ml of water sample. The following formula was used:

Viable bacterial count (CFU/ml) = Number of colonies on agar plate × dilution factor × 10

After calculations, the average values (CFU/ml) were calculated for each water subsample for statistical analysis and plotting.

### Determination of physicochemical parameters

As described in Hossain et al. [8], the physicochemical parameters such as temperature, DO, pH, and NH_3_ were determined using a glass thermometer (Saraan, Denmark), DO test kit (Biosol, AA Biotech, India), pH test kit (CP-102, Thailand), and Ammonia Test Kit (AQUA AM), respectively, as described in Hossain et al. [8]. After 1 min, the water temperature was measured by inserting the end bulb of a glass thermometer (Saraan, Denmark) into the water and recording the temperature in degrees Celsius (°C).

The water’s DO content was obtained as mg/l by the DO test kit (Biosol, AA Biotech, India). The DO was required to be fixed before testing. The DO test bottle was rinsed 2–3 times with sample water. It is necessary to avoid any air bubbles inside the bottle. For this reason, the water was filled until it overflowed and stopped immediately. From the test kits, DO-1 and DO-2 reagents were taken, and the water samples were treated (10 drops of DO-1 and then 10 drops of DO-2), waited for 1 min, brown precipitation developed and allowed to settle. After resting the test bottle for a minimum of 20 min, 10–20 drops of reagent DO-3 were mixed and shaken well to dissolve the precipitation. The above-treated sample was then used for testing.

For this reason, 10 ml of fixed sample was added to the test jar, four drops of DO-4 reagent were added, and the mixture was well mixed. Then, the DO-5 reagent was added dropwise. The number of drops was counted until the blue color disappeared. Finally, the DO content was calculated using the following formula:

DO (mg/l) = 0.65 × (No. of drops of DO-5 reagent)

The pH of the collected water was measured by the pH test kit (CP-102, Thailand). A supplied color cell was initially cleaned with the collected water sample. The color cell was then filled with 5 ml of the same water sample, to which two drops of pH reagents were added. After mixing well, the generated color was matched with the supplied standard color scale to obtain a pH value.

An ammonia test kit (AQUA AM) was used to determine the NH_3_ concentration of the collected water. For this purpose, the supplied measuring vessel was cleaned with the test water sample. The vessel was then filled with water up to the 5 ml mark with the test water. The water was treated with two drops of the Ammonia-1 reagent and mixed well. Then two drops of ammonia-2 reagent followed by two drops of ammonia-3 reagent were added and thoroughly mixed. At the final step, 4 drops of ammonia-4 reagent were thoroughly mixed. The mixture was then rested for 20 min. The top view color of the measuring vessel was compared with the supplied color band to obtain a read of the NH_3_ concentration.

### Statistical analysis

Collected data were recorded, and some analyses were done in MS Excel 2010. Analysis of variance was carried out at a 5% (*p *< 0.05) significance level, and Duncan’s New Multiple Range Test was employed with IBM Statistical Package for the Social Sciences Statistics (Version-22.0).

## Results

### Distance and duration of the supply channels

From the point of origin of the supply channel to the final destination, the distance was about 140, 200, and 305 km, respectively, for supply channels 1, 2, and 3. The time required to transport the fish to the designated unloading points was 6, 8, and 8 h in supply channels 1, 2, and 3, respectively. The required time was not entirely relevant to the distance, but rather the road conditions and traffic jams.

### Changes in the viable count of bacteria

Changes in viable counts of bacteria in different supply channels are compared in Table 1. Viable bacterial counts increased significantly (p < 0.05) at 2 h compared to 0 h in all the sampling channels. Again, bacterial counts were found to increase gradually with the length and traveling time of the transportation route, though it was insignificant. In all the sampling channels, viable counts decreased at hour 4, but it was significant only in the case of channel 3 (Table 1).

At 0 h, the highest viable count of bacteria was 0.14 ± 0.02 × 10^5^ CFU/ml in supply channel 2, which significantly increased to 3.70 ± 1.33 × 10^5^ CFU/ml after 2 h, and finally, became 3.36 ± 1.38 × 10^5^ CFU/ml after 8 h of transportation. The lowest initial (at 0 h) viable bacterial count was found in the case of supply channel 1 (0.08 ± 0.01 × 10^5^ CFU/ml), which significantly increased to 3.08 ± 0.97 × 10^5^ CFU/ml after 2 h, and finally, became 2.85 ± 0.32 × 10^5^ CFU/ml after 6 h. In supply channel 3, bacterial count significantly increased from the initial value, 0.09 ± 0.01 × 10^5^ CFU/ml, to a maximum of 3.61 ± 1.42 × 10^5^ CFU/ml after 8 h of transportation. Although viable bacterial count was increased with time during transportation of Pangasius catfish, a fall in counts was observed in all the sampling channels after 4 h of transportation (Table 1).

### Changes in physicochemical parameters

**Figure 2.**
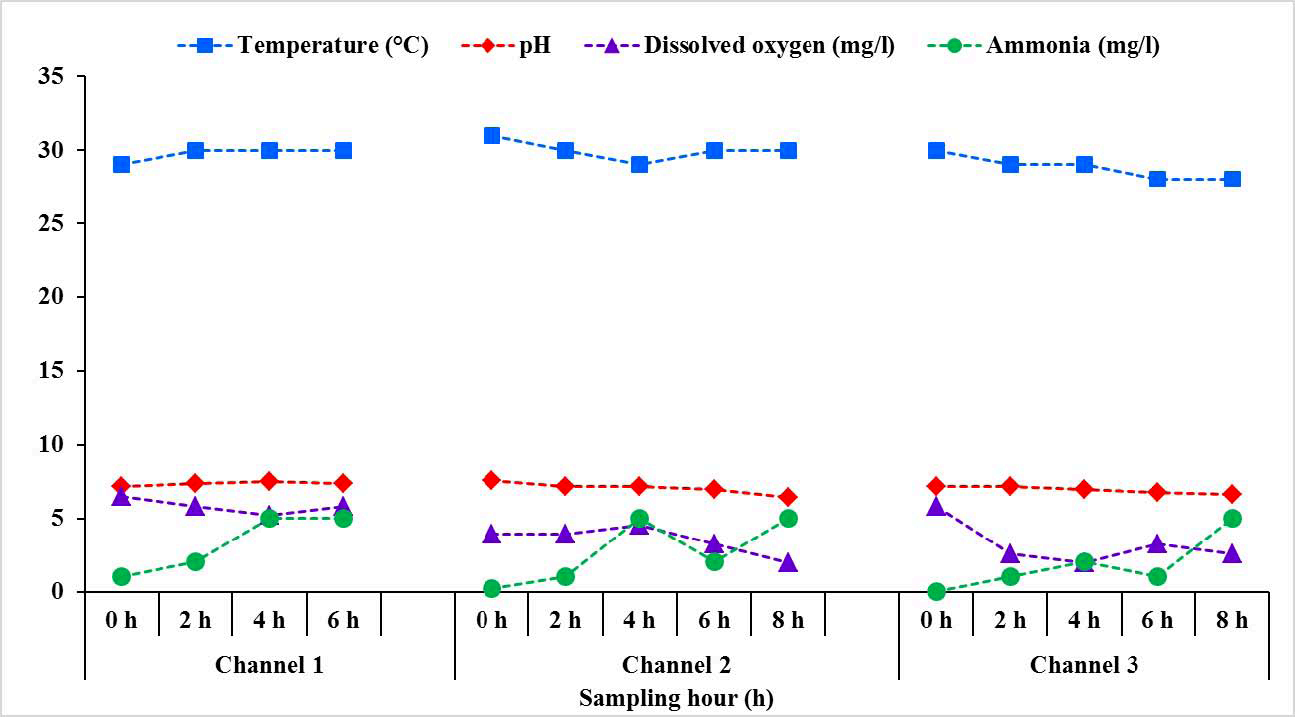
Changes in physicochemical parameters of water subsamples collected from different supply channels of live transportation of Pangasius catfish (*P. hypophthalmus*) in Bangladesh.

Changes in physicochemical parameters of transporting water collected from different supply channels of live Pangasius catfish are shown in Figure 2. Among the physicochemical parameters, there was a little change in temperature from the start of transportation to the end of all three sampling supply channels. The temperature ranged from 29 to 30 in supply channel 1, while it was 29 to 31 in supply channel 2, and 28 to 30 in supply channel 3. DO and pH dropped gradually with the duration of transportation, while the NH_3_ concentration in water increased gradually over time. The highest initial DO was seen in channel 1 (6.5 mg/l) that finally dropped to 5.2 mg/l at the period of unloading in the destination (Dhaka). While the lowest initial DO value was 3.9 mg/l in supply channel 2, which declined to 1.95 mg/l after 8 h of transportation. Compared to the values of previous subsamples, the levels of DO was found to increase slightly at 6 h in the case of supply channels 1 and 3, and at 4 h at supply channel 2.

The pH values of the subsamples at 0 h ranged between 7.2 and 7.6 and slightly dropped to 6.4 in the case of supply channel 2 and 6.6 in the case of supply channel 3 at the end (8 h) of transportation. However, the pH values were almost stable in the case of channel 1. Regardless of the distance and duration of the supply channels, gradual increases in NH_3_ concentrations were observed in all of the three supply channels. At 0 h, NH_3_ concentrations were 1, 0.2, and 0 mg/l in supply channels 1, 2, and 3, respectively. The concentrations of NH_3_ were then increased to 5 mg/l in all the supply channels. However, a slight fall from 5 to 2 mg/l and 2 to 1 mg/l was observed at 6 h in supply channels 2 and 3, respectively.

**Table 1.** Bacterial viable counts in water samples collected from different supply channels during live transportation of Pangasius Catfish (*P. hypophthalmus*) in Bangladesh.

Time (h)	Bacterial viable counts (CFU/ml) × 10^5^		
Channel 1	Channel 2	Channel 3
0	0.08 ± 0.01^b^	0.14 ± 0.02^b^	0.09 ± 0.01^b^
2	3.08 ± 0.97^a^	3.70 ± 1.33^a^	2.87 ± 1.10^a^
4	2.60 ± 0.87^a^	2.38 ± 1.13^a^	1.81 ± 0.74^ab^
6	2.85 ± 0.32^a^	2.83 ± 1.16^a^	3.30 ± 1.36^a^
8	No data	3.36 ± 1.38^a^	3.61 ± 1.42^a^

Mean values with the same superscript letter(s) were not significantly different (*p* > 0.05).

### Relationship among viable bacterial counts and physicochemical parameters

**Figure 3.**
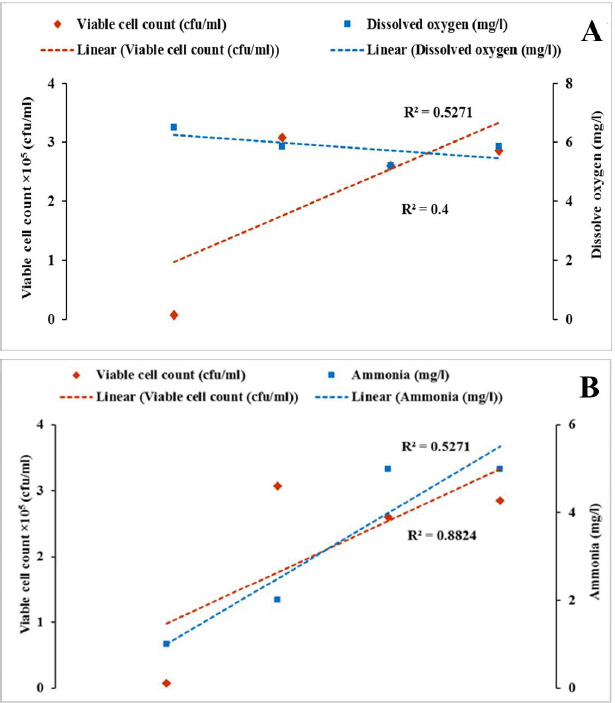
Correlation between (A) Viable count of bacteria and DO and (B) Viable count of bacteria and NH_3_ in supply channel-1 during live transportation of live Pangasius catfish (*P. hypophthalmus*) in Bangladesh.

**Figure 4.**
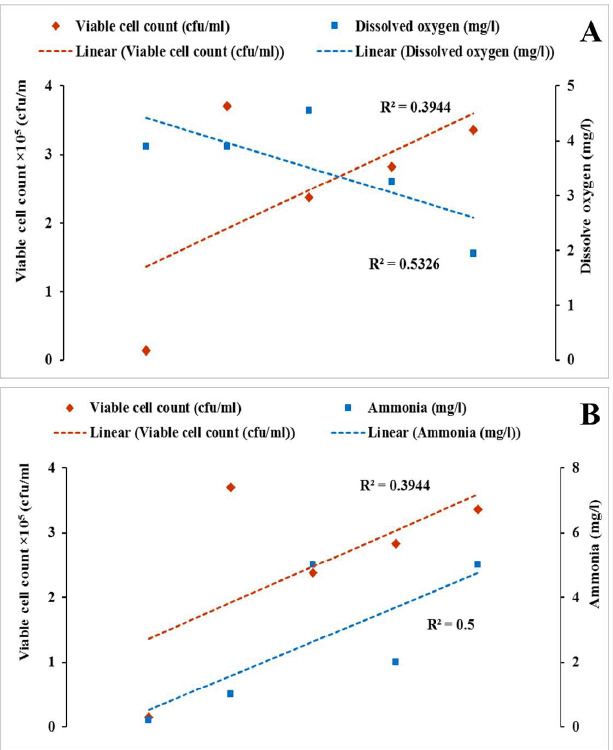
Correlation between (A) Viable count of bacteria and DO and (B) Viable count of bacteria and NH_3_ in supply channel-2 during live transportation of Pangasius catfish (*P. hypophthalmus*) in Bangladesh.

**Figure 5.**
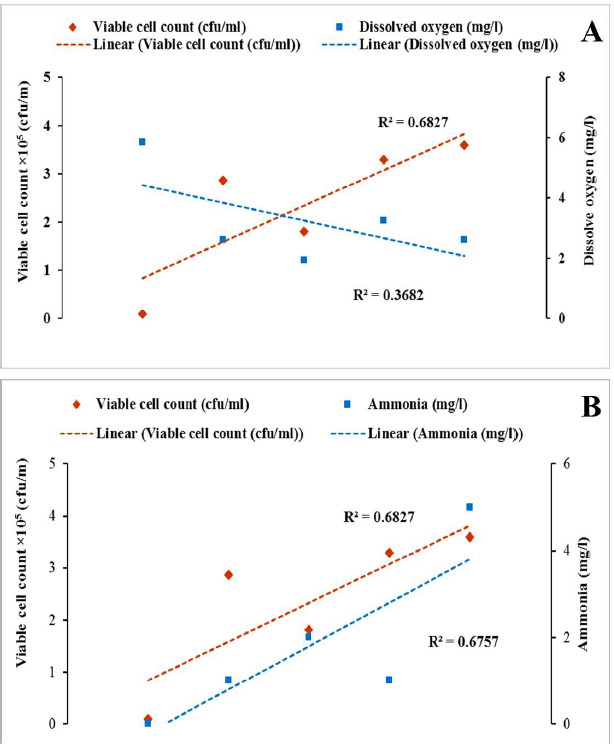
Correlations between (A) Viable count of bacteria and DO and (B) Viable count of bacteria and NH_3_ in supply channel-3 during live transportation of Pangasius catfish (*P. hypophthalmus*) in Bangladesh.

In this study, the DO content of water decreased, and the NH_3_ concentration increased with time during the live transportation of Pangasius catfish (Fig. 2). The viable bacterial counts in the transportation water were also raised with transportation periods (Table 1). Figures 3–5 show the patterns of changes in viable counts of bacteria, DO levels, and NH_3_ concentrations with time. It was found that the viable counts of bacteria were negatively correlated with DO levels of transport water (Figs. 3A, 4A, and 5A) and positively correlated with NH_3_ concentrations (Figs. 3B, 4B, and 5B). Analysis of correlation (Pearson) between the viable bacterial count and water parameters was conducted at a 5% level of significance (p < 05), and the values are presented in (Table 2). A moderate positive correlation (*r* = 0.638) was observed between the viable bacterial count and NH_3_ in supply channel 1. In contrast, a moderate negative correlation (*r* = −0.737) was observed between viable bacterial count and DO in the same supply channel (Table 2, Fig. 3). In the case of supply channel 2, a weak negative correlation (*r* = −0.362) was observed between viable bacterial counts and the DO level of water. In contrast, a moderately positive correlation (r = 0.422) was observed between viable counts and NH_3_ concentration (Table 2, Fig. 4). The viable bacterial count showed a moderate negative correlation (*r* = −0.727) with the DO level and a moderate positive correlation (*r* = 0.620) with the NH_3_ concentration of water subsamples from supply channel 3 (Table 2, Fig. 5).

## Discussion

Live transportation of Pangasius catfish (P. hypophthalmus) has commercial importance to the transporters and the sellers of the final consumer market. The adverse state of physicochemical parameters and bacterial counts made the Pangasius catfish less lucrative to consumers, ultimately affecting the fish’s market value. This study followed changes in the viable bacterial count and physicochemical parameters in the water used during live transportation of Pangasius catfish, and the correlations in their changing patterns were established. At every 2-h interval, subsamples of water were collected. Water samples were diluted and plated onto agar plates with replications for the viable bacterial count. Water temperatures, DO, pH, and NH_3_ concentration of the collected subsamples were also measured. It was assumed that the viable count of the bacteria would gradually increase with transportation periods because of their regrowth in the water and show a positive correlation with NH_3_ concentration. We also hypothesized that accumulation and decomposition of the metabolic wastes would decrease the pH and DO levels and thus show a negative correlation with viable counts. 

In this study, subsamples from all three supply channels showed a gradual increase in viable bacterial counts (Table 1). Some previous research also reported increased viable counts of bacteria with time during the live transportation of fish [9,14,15]. As a common practice, deep tube well water was used during the live transportation of Pangasius catfish. Islam et al. [16] stated that the bacterial load in deep tube well water is very low. Thus, the presence and gradual increase in the viable counts of bacteria in all three supply channels may be due to several reasons. First, the microbial composition of the live fish reflects the microbial content of their habitat. They can also be transmitted from the fish to the surrounding waters [17]. In general, fish skin contains bacteria ranging from 10^2^–10^7^ CFU cm^−2^ and fish gills and intestines from 10^3^–10^9^ CFU gm^−1^ [18]. Second, the excretory organic materials accumulated in the tank’s waters were decomposed and utilized by the bacteria, resulting in their regrowth. Third, while transporting fish in live conditions, one of the greatest challenges is maintaining a suitable range of physicochemical parameters to reduce the intensity of the stress on fish and/or their mortality rate. Due to stress, many fish die before reaching the desired market during live transportation. Previous research has found that dead fish in water increases the bacterial population [9]. Moreover, the water temperature was nearly stable, close to 30°C in all studied channels. It provided a suitable environment for bacteria, increased their metabolic activities, and facilitated their higher growth [19]. This study also showed a decrease in the bacterial count after 4 h. This may be due to partial replacement of tank water with pressurized deep tube well water after 2–3 h of transportation, which has been done in the country during live transportation of Pangasius catfish.

Results also showed that the water temperatures were almost stable regardless of the supply channels and transportation period at around 30°C. However, the DO concentration and pH level decreased gradually, but the NH_3_ concentrations of the water increased in all three sampling channels (Fig. 2). Previous reports also showed a limited water temperature fluctuation during the transportation of live fish [20]. Bui et al. [20] reported a slight change in water temperature during live transportation of striped catfish. Although it was reported that the changing pattern of temperatures during live transport of fish in tropical conditions probably impose minimum to no stress effects on them [21], the temperature is the regulator of so many other circumstances in water. Temperature increases the metabolic activities of aquatic micro, and macro-organisms resulting in depletion of DO in water, becoming a critical factor for organisms to survive. High temperatures also increase the rate of degradation of the organic substances, depleting the level of O2 in water [22]. Temperature also controls the holding capacity and mixing of the gaseous substances and minerals in the water, maintains the physicochemical properties, and regulates the pH level of the water [23]. The pH of water is another vital water quality parameter for aquatic organisms for their growth and survival. In aquatic environments, almost all of the chemical reactions are controlled by water pH levels. The pH values are found to fluctuate during live transportation of fish [20,24], similar to our findings. Previous research also reported a decreasing trend of water pH during live transportation fishes [25–27]. This study showed that the pH values were decreased from the initial levels with time, especially after 8 h, in all three supply channels (). It was expected that the metabolic activities of the live fishes (Pangasius catfish) as well as the bacteria in the tank waters resulted in an increased accumulation of CO2 in the water, which acidifies the water, and the pH values were reduced. Nevertheless, the pH range was within the acceptable level (ranged from 6.4 to 7.6 within the studied channels) for the fishes to survive (Fig. 2).

In all the studied supply channels, the DO levels of the subsamples were found to decrease gradually and, in some cases, reach a critical level, especially after 8 h (Fig. 2). Previous research also showed similar fluctuations and gradual depletion of DO levels over transportation [9,20]. It was also reported that the first 30–60 min of transporting live fish are critical [28], with a reduction in DO level of up to 5 mg/l observed during this time [29]. DO is a limiting factor for fish to survive in different water quality parameters. The gradual reduction of DO levels in water during the transportation is not difficult. The fish in the tanks were stocked at a higher density, accelerating their metabolic activities and oxygen consumption. It was also mentioned that increased decomposition and oxidation rates of organic matters (e.g., accumulated metabolic wastes in the fish transportation tanks) by microorganisms depleted the DO while reducing the solubility of oxygen in water [22].

Several factors (stressors) create pressure on the fish during live transportation, and the effects and nature of these stress-inducing factors vary. Stressors have been summarized into three basic groups by Barton [30], of which physical (such as handling, transportation, etc.) and chemical stressors (such as low oxygen concentration, increased acidity, etc.) are crucial. The chemical stressors are believed to affect the fish’s welfare during live transportation directly. However, it should be noted here that although the DO levels were reduced to the critical level during the live transportation of Pangasius catfish, the fish mostly survived. This may be because of the introduction of oxygen from the atmosphere due to water-air interactions, especially at the surface. Moreover, Pangasius catfish have a swimbladder as an accessory respiratory organ that allows them to survive even in that critical condition [31]. In this study, compared to the values of previous subsamples, a slight surge in O2 levels was observed at 6 h in the cases of supply channels 1 and 3, and at 4 h in supply channel 2. This would be due to the slight agitation of the transportation tank and the partial replacement of water with pressurized deep tube well water during transportation.

The concentrations of NH_3_ were found to increase and reach 5 mg/l in all three studied channels at the end of the transportation period (6–8 h). However, a decrease in NH_3_ concentration was observed at 6 h of transportation in supply channels 2 and 3 (Fig. 2). Previous studies also reported an increase in the NH_3_ concentration during the live transportation of fish [5,27]. Similar results regarding the changes in NH_3_ concentration during the live transportation of Pangasius catfish are also reported by Dhanasiri et al. [32] and Bui et al. [20]. The decreased concentration of NH_3_ at 6 h () may be due to the partial exchange of water on the way during transportation. Metabolic nitrogenous end-products, including NH_3_, are produced by fish due to the catabolism of proteins and/or other nitrogenous chemical compounds. The fish then excrete the NH_3_ into the surrounding waters. During the live transportation of fish, an accumulation of NH_3_ in a fixed volume of water occurs, ultimately resulting in a gradual increase in its concentration. Furthermore, the NH_3_ concentration in transport water may rise as a result of bacterial action and decay of accumulated waste materials such as leftover feed, feces, dead micro-and macro-plants in water, and so on [23,33]. During live transportation of fishes, due to the transportation-related stresses, increased NH_3_ concentration and decreased levels of DO led to the death of some fishes [34]. For longer durations and distances, partial degradation or deterioration of these dead fishes due to the action of self-enzymes and bacteria may also contribute to the generation of NH_3_.

**Table 2.** Correlations between the viable count of bacteria and physicochemical parameters (DO level and NH_3_ concentration) in different supply channels during live transportation of Pangasius Catfish (*P. hypophthalmus*) in Bangladesh.

	Channel 1				Channel 2			Channel 3		
		Viable count	NH_3_	DO	Viable count	NH_3_	DO	Viable count	NH_3_	DO
Viable count	Pearson Correlation	1	0.638^a^	−0.737^a^	1	0.422^a^	−0.362^a^	1	0.620^a^	−0.727^a^
	Sig. (2-tailed)		0.362	0.263		0.479	0.549		0.264	0.164
	*N*	4	4	4	5	5	5	5	5	5
NH_3_	Pearson Correlation	0.638^a^	1	−0.792	0.422^a^	1	−0.299	0.620^a^	1	−0.554
	Sig. (2-tailed)	0.362		0.208	0.479		0.625	0.264		0.332
	*N*	4	4	4	5	5	5	5	5	5
DO	Pearson Correlation	−0.737^a^	−0.792	1	−0.362^a^	−0.299	1	−0.727^a^	−0.554	1
	Sig. (2-tailed)	0.263	0.208		0.549	0.625		0.164	0.332	
	*N*	4	4	4	5	5	5	5	5	5

*N* = number of cases used.

a Correlation is significant at the 0.05 level (2-tailed).

Despite the production and accumulation of NH_3_ during the live transportation of fish, most of the Pangasius catfish were found to survive in this study. This may be because of the higher mixing and possible dissolution of the excreted or produced CO2 and NH_3_. These gases tend not to accumulate in nature or under typical cultural conditions. In aqueous situations, the majority of the NH_3_ gas dissolves in water to become a nontoxic ammonium ion (NH_4_^+^), and the CO2 is dissolved to become mild carbonic acid [35]. Furthermore, because the Pangasius catfish is an air-breathing fish [31], it may have mechanisms to cope with and/or withstand the toxic effects of NH_3_ [36]. Despite most fishes surviving in this study, higher NH_3_ concentrations may lead to NH_3_ toxicity in fish transport, especially during long hauls. Ross and Ross [13] described how increased CO2 and NH_3_ concentrations could result in a higher death rate of fish if these gases excessively dissolve and accumulate in the transport water to the point of becoming toxic to the live fish. Higher concentrations of NH_3_ in water increase its concentration in the plasma of fish (Cardinal tetra, *Paracheirodon axelrodi*), depending on transport time and fish bulkiness [37,38].

Previous research reported that higher NH_3_ levels create adverse situations and induce injurious conditions such as disruptions in osmoregulation activities, damage to the kidneys, and branchial epithelium, etc. Increased concentrations of NH_3_ are believed to deteriorate the oxygen intake capacity of the gills and oxygen transport capacity of the blood due to deviations in the normal pH level of the blood. These consequences damage blood cells, making it harder for fish to respire [39]. Moreover, increased NH_3_ concentration negatively affects other water quality parameters. High NH_3_ (also known as NH_4_^+^) concentrations can directly impact the DO levels of water, as about 4.3 mg of oxygen is necessary to oxidize 1.0 mg of NH_4_^+^ [34,40]. An increased concentration of NH_3_ also elevates the pH (and a reduction of the pH will, therefore, reduce NH_3_ toxicity) and vice-versa [41]. In our study, viable bacteria counts were negatively correlated with DO levels of transport water (Figs. 3A, 4A, and 5A), while they were positively correlated with NH_3_ concentrations (Figs. 3–5). Previous studies also reported similar negative correlations between viable bacterial counts and DO [42,43] and positive correlations between viable bacterial counts and NH_3_ [43,44] in water. As previously discussed, transport water may receive more bacteria from the fish itself and can be increased through its regrowth. At a suitable range of water temperatures, bacteria utilize the accumulated organic matter (excretory materials, uneaten feeds, etc.), and dead organisms facilitate their regrowth. This increased rate of metabolic activity of aquatic micro (bacteria) and macro (fish itself) exhausts the DO in water, resulting in a negative correlation between viable counts of bacteria and the DO level of water. On the other hand, NH_3_ is produced by the fish due to the catabolism of proteins and/or other nitrogenous chemical compounds and excreted into the surrounding waters. The NH_3_ concentration in transport water increases due to the bacterial action and decomposition of accumulated organic materials. This results in a positive correlation between viable counts of bacteria and the NH_3_ concentration of transport water used during the live transportation of Pangasius catfish. 

## Conclusion

It can be concluded that gradual changes in viable counts of bacteria and physicochemical parameters with the duration of transportation indicate an unfavorable environment for the survival of Pangasius catfish. An increase in the bacterial population competes with fish for DO and promotes degradation of excretory substances to heighten NH_3_ concentrations, ultimately making live transportation challenging.

## List of Abbreviations

CFU, colony-forming unit; DO, Dissolved oxygen; h, Hour; mg/l, Milligram per liter.
